# Primary fibroblasts from CSPα mutation carriers recapitulate hallmarks of the adult onset neuronal ceroid lipofuscinosis

**DOI:** 10.1038/s41598-017-06710-1

**Published:** 2017-07-24

**Authors:** Bruno A. Benitez, Mark S. Sands

**Affiliations:** 1 Department of Medicine, Washington University School of Medicine, St. Louis, MO 63110 USA; 2 Department of Genetics, Washington University School of Medicine, St. Louis, MO 63110 USA; 3 Hope Center for Neurological Disorders, Washington University School of Medicine, St. Louis, MO 63110 USA

## Abstract

Mutations in the co- chaperone protein, CSPα, cause an autosomal dominant, adult-neuronal ceroid lipofuscinosis (AD-ANCL). The current understanding of CSPα function exclusively at the synapse fails to explain the autophagy-lysosome pathway (ALP) dysfunction in cells from AD-ANCL patients. Here, we demonstrate unexpectedly that primary dermal fibroblasts from pre-symptomatic mutation carriers recapitulate *in vitro* features found in the brains of AD-ANCL patients including auto-fluorescent storage material (AFSM) accumulation, CSPα aggregates, increased levels of lysosomal proteins and lysosome enzyme activities. AFSM accumulation correlates with CSPα aggregation and both are susceptible to pharmacological modulation of ALP function. In addition, we demonstrate that endogenous CSPα is present in the lysosome-enriched fractions and co-localizes with lysosome markers in soma, neurites and synaptic boutons. Overexpression of CSPα wild-type (WT) decreases lysotracker signal, secreted lysosomal enzymes and SNAP23-mediated lysosome exocytosis. CSPα WT, mutant and aggregated CSPα are degraded mainly by the ALP but this disease-causing mutation exhibits a faster rate of degradation. Co-expression of both WT and mutant CSPα cause a block in the fusion of autophagosomes/lysosomes. Our data suggest that aggregation‐dependent perturbation of ALP function is a relevant pathogenic mechanism for AD-ANCL and supports the use of AFSM or CSPα aggregation as biomarkers for drug screening purposes.

## Introduction

The Neuronal Ceroid Lipofuscinoses (NCLs, also referred as Batten’s disease) are the most common (~1 in 12,500 births) inherited childhood neurodegenerative diseases^1^. Clinical symptoms and neuropathological changes appear over a wide range of age from birth to early adulthood. The intracellular accumulation of autofluorescent storage material (AFSM) regardless of the disease-causing protein or its subcellular localization is the hallmark of NCL.

Autosomal dominant, adult-onset neuronal ceroid lipofuscinosis (AD-ANCL) (MIM #162350) is a rapidly progressive neurodegenerative disease characterized by early onset dementia, seizures, motor impairment and is invariably fatal^2, 3^. AD-ANCL is caused by a single-nucleotide variation (c.344 T > G) or an in-frame single codon deletion (c.346_348 delCTC) in one allele of the *DNAJC5/NCL4B* gene^4–6^. The *DNAJC5* gene encodes cysteine string protein alpha (CSPα). Currently, the effect of mutations in CSPα on lysosome function and accumulation of AFSM remains to be clarified. Most known CSPα functions are due to its co-chaperone or chaperone activity. However, AD-ANCL-causing mutations (p.L115R or p.L116del) are located in the cysteine string domain^5, 6^ and both retain chaperone activity^7^. For the last 20 years, studies primarily in neurons from CSPα-deficient mice have suggested that CSPα chaperone function is exclusively located at the synapse^8–10^. However, CSPα has been found in lysosome-enriched fractions^11–14^ and involved in the pathogenesis of Lysosomal storage diseases (LSDs)^14, 15^. Furthermore, a recent proteomic analysis of brain tissue from terminal AD-ANCL patients revealed significant changes in lysosomal proteins rather than synaptic proteins^16^. In addition, a brain from a pre-symptomatic CSPα mutation carrier revealed that accumulation of AFSM and lysosome dysfunction precedes synaptic degeneration^17^. These results question the current dogma about the exclusive synaptic function of CSPα.

The cellular system mediating CSPα degradation is currently unknown. A recent report suggests that CSPα is degraded mainly by the ubiquitin-proteasome system (UPS)^15^. However, the UPS degrades mainly short-lived proteins and CSPα is a long-lived protein^18^. In addition, there is no canonical ubiquitylation site in CSPα^19^ and proteasome inhibitors have had no effect on CSPα levels in different cell types^20, 21^. Brain tissue from terminal AD-ANCL cases exhibit a significant reduction in the levels of CSPα^5, 22, 23^. It is not clear if, or how mutant CSPα reduces the levels of wild-type CSPα in AD-ANCL patients^23, 24^.

Mutant CSPα is more hydrophilic than wild-type and acquires an intrinsic propensity to self-assemble into aggregates^17^. Mutant and wild type CSPα interact to form high molecular weight aggregates^7, 22, 25^. However, the link between aggregates, AFSM and lysosomes is still unclear.

Here, we demonstrate that primary dermal fibroblasts from asymptomatic mutation carriers recapitulate features of AD-ANCL *in vitro* including AFSM accumulation, CSPα-p.L115R/CSPα-WT aggregates and the structural and functional lysosomal dysfunction found in the brains of AD-ANCL patients. We show that the levels of CSPα-p.L115R/CSPα-WT aggregates correlate with accumulation of AFSM and both are susceptible to pharmacological intervention *in vitro*. In summary, these data suggest a novel role of CSPα in lysosome physiology, a mechanistic link between AFSM and CSPα-p.L115R/CSPα-WT aggregates and a potential new treatment for AD-ANCL through the modulation of the ALP.

## Results

### Lysosome dysfunction in AD-ANCL *in vivo* and *in vitro*

**Figure 1:**
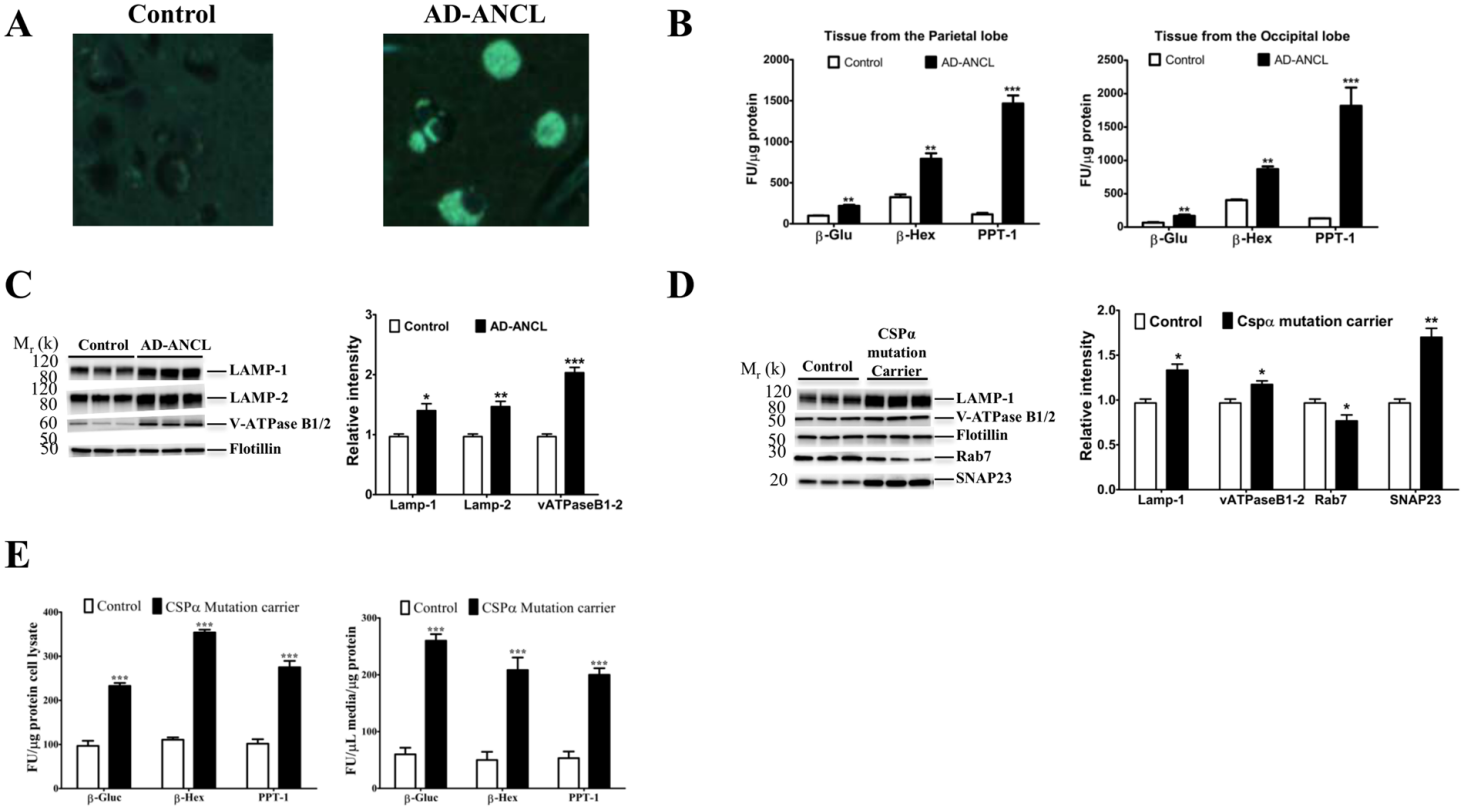
Lysosome dysfunction in AD-ANCL *in vivo* and *in vitro*. (**A**) Representative images of AFSM in cortical pyramidal neurons from an AD-ANCL patient (right *panel*) and control (*left panel*). (**B**) Graph shows the lysosomal enzyme activities of PPT-1, β-gluc and β-Hexa measured in the parietal lobe and occipital lobe from three AD-ANCL patients compared to the same brain regions from three controls. Enzymatic activity was normalized to the total protein and pooled by genotype. (Mean ± SEM of quantification by triplicate in each individual). **p ≤ 0.01; ***p ≤ 0.001. (**C**) Representative Western blots showing the expression of LAMP-1, LAMP-2 and V-ATPase B1/2 in occipital lobe from three controls and three AD-ANCL patients. Transmembrane proteins are normalized to Flotillin. The histogram shows the quantification of LAMP-1, LAMP-2 and V-ATPase B1/2 detected by immunoblot relative to control levels. (**D**) Representative Western blots illustrate the expression of LAMP-1, V-ATPase B1/2, Rab7 and SNAP23 in fibroblasts from controls and asymptomatic CSPα mutation carriers. Transmembrane proteins are normalized to Flotillin. The histogram shows the quantification of LAMP-1, V-ATPase B1/2, Rab7 and SNAP23 detected by immunoblot relative to control levels. (**E**) Graph shows the lysosomal enzyme activities of PPT-1, β-gluc and β-Hexa in the culture medium (*right panel*) or in the cell homogenates (*left panel*) of two CSPα mutation carriers relative to cells from two age-matched control individuals. Enzymatic activity was normalized to the total intracellular protein and pooled by genotype. (Mean ± SEM of quantification by triplicate in each individual). **p ≤ 0.01; ***p ≤ 0.001.

Post-mortem analysis of the brains of AD-ANCL patients reveals marked enlargement of the cortical pyramidal neurons containing typical AFSM compared to a control sample (Fig. 1A). In addition, two different brain regions from three terminal AD-ANCL patients revealed significant secondary elevations (p ≤ 0.01) in the activity of the lysosomal enzymes palmitoyl-protein thioesterase 1 (PPT-1), β-glucuronidase (β-gluc,), and β-hexosaminidase (β-Hexa) (Fig. 1B). The levels of membrane-associated lysosomal proteins LAMP-1, LAMP-2 and V-ATPase B1/2 (Fig. 1C), and intraluminal proteins including Saposin D and PPT-1 are increased in AD-ANCL patients in the occipital lobe (Supplementary Fig. 1A). There is an increase in both transmembrane and soluble lysosomal proteins across different brain regions (frontal, parietal, temporal and cerebellum) among the AD-ANCL patients (Data not shown). Unexpectedly, cultured fibroblasts from CSPα mutation carriers display significantly increased levels of V-ATPase B1/2, LAMP-1 (Fig. 1D), and intraluminal Saposin D proteins (Supplementary Fig. 1B). In addition, there is an increase in SNAP23 levels and reduced levels of Rab7 in fibroblasts from CSPα mutation carriers (Fig. 1D). There is also intracellular elevations (p ≤ 0.01) in β-Hexa, PPT-1 and β-gluc activities (Fig. 1E). Interestingly, the levels of lysosomal enzymes were elevated in the medium to a greater extent than in the cells themselves (Fig. 1E).

### There is a correlation between AFSM accumulation and CSPα.pL115R aggregation

**Figure 2:**
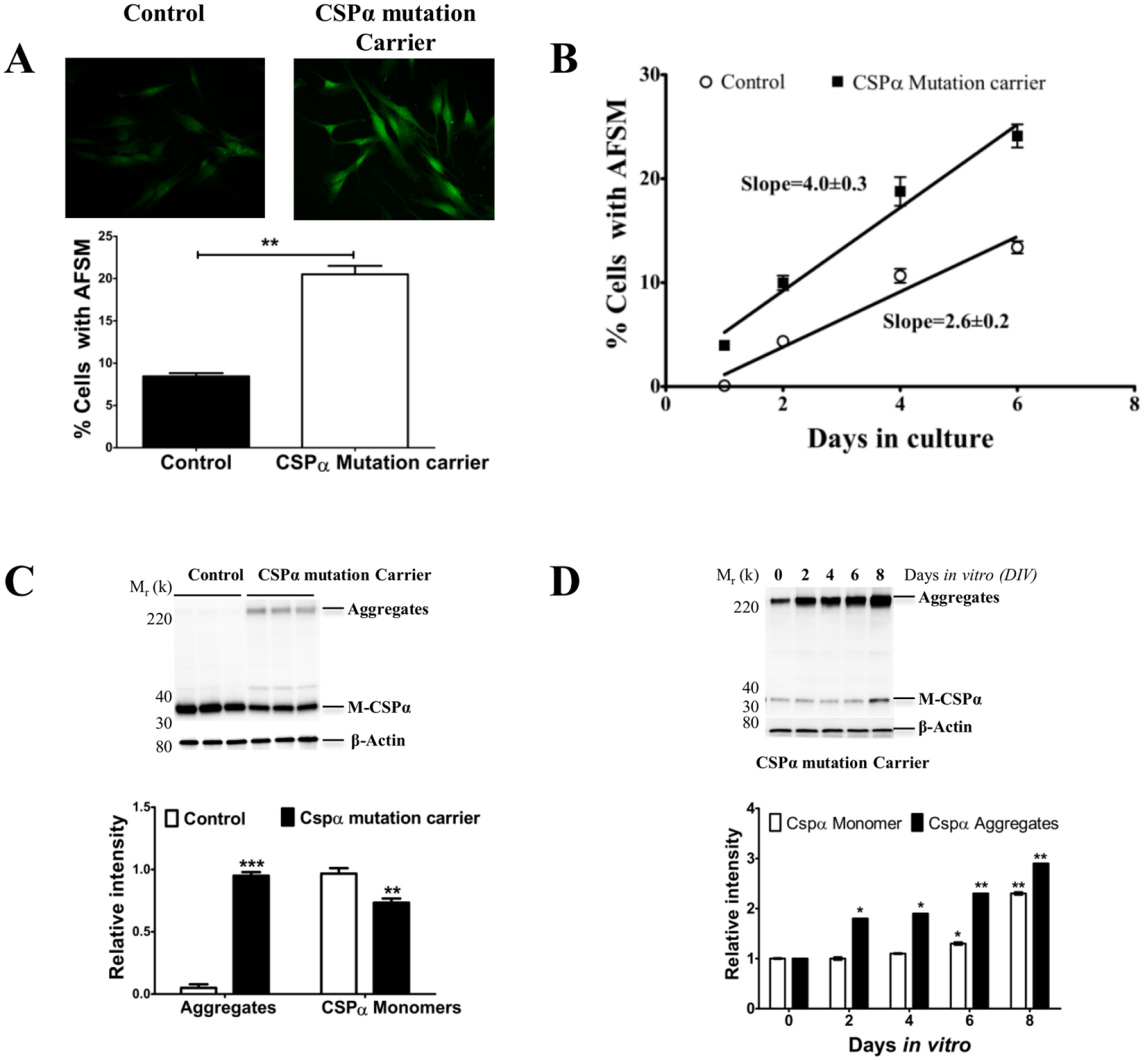
Time course of AFSM and CSPα.pL115R aggregate accumulation *in vitro*. (**A**) Representative images of AFSM in primary dermal fibroblasts from an asymptomatic CSPα mutation carrier (*top, right panel*) and control (*top, left panel*). The histogram shows the quantitative analysis of AFSM by flow cytometry. (**B**) The rate of AFSM accumulation (calculated as the slope of the percentage of cells with autofluorescence). The data points were fitted using linear regression analysis. (**C**) Representative Western blot showing the expression of CSPα monomers (M-CSPα) and its aggregates (Aggregates) in human fibroblasts from CSPα mutation carriers and controls. The histogram shows the quantification of CSPα monomers (M-CSPα) and CSPα aggregates (Aggregates) detected by immunoblot relative to control levels. Proteins are normalized to β-actin. (**D**) Representative Western blot showing the levels of CSPα monomers (M-CSPα) and CSPα aggregates (Aggregates) after 8 days in culture normalized to β-actin. The histogram shows the quantification of CSPα monomers (M-CSPα) and CSPα aggregates (Aggregates) detected by immunoblot relative to protein levels on day zero. Proteins are normalized to β-actin.

Fibroblasts from human CSPα-p.L115R carriers exhibit a two-fold increase in AFSM (median fluorescence intensity [mfi]) compared to cells from age-matched controls (Fig. 2A). There is also a six-fold (0.67% vs. 3.89%) increase in the number of cells with AFSM in fibroblasts from asymptomatic CSPα mutation carriers (Fig. 2A). There are progressive elevations in AFSM, from 1.7 to 2.1-fold (n = 8, p = 0.002) as the cells age *in vitro* (Fig. 2B). The differences in AFSM accumulation are attributed mainly to the days in culture (67.8% of the total variation, p = 0.0001) but, are also due to the genotype (26.6% of the total variation, p = 0.0001). The rate of AFSM accumulation (calculated as the slope of the percentage of cells with autofluorescence higher than average) is 1.5-fold faster in CSPα-p.L115R carriers than in controls (Fig. 2B). There is a reduction in the level of CSPα monomers and an accumulation of CSPα aggregates compared to age-matched controls (Fig. 2C). Fibroblasts from asymptomatic CSPα mutation carriers exhibit a time-dependent increase in the levels of mutant CSPα aggregates (Fig. 2D).

### CSPα and its aggregates are located to the lysosome

**Figure 3:**
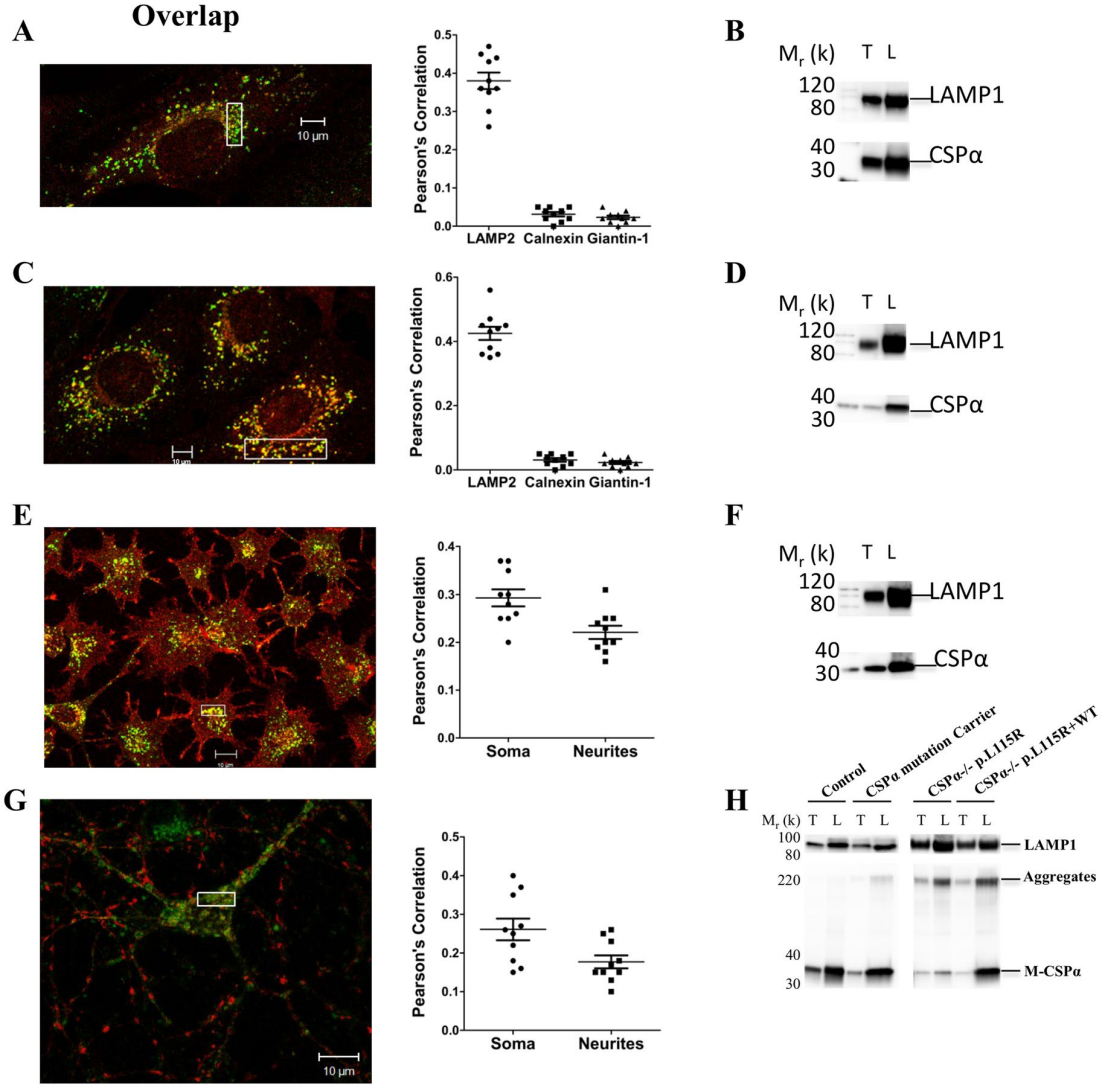
CSPα and its aggregates localize to the lysosome. (**A**) Representative high magnification merged (*overlap, yellow*)﻿ pictures of normal human primary fibroblasts stained for endogenous CSPα (CSPα, red) and LAMP-2 (LAMP-2, green). Graph shows the co-localization index (Pearson correlation) between the green (LAMP-2) and the red (CSPα) channels within the boxed area. The graph also shows the Pearson correlation between (*CSPα, red*) and Calnexin (*green*) and Giantin-1 (*green*) (See Supplemental Fig. 1C,D). (**B**) Representative Western blot of LAMP-1 and CSPα from the total cell homogenates (T) and lysosome enriched (L) fractions of normal human fibroblasts. (**C**) Representative high magnification merged (*overlap, yellow*) pictures of murine primary fibroblasts stained for endogenous CSPα (*CSPα, red*) and LAMP-2 (*LAMP-2, green*). Graph shows the co-localization index (Pearson correlation) between the green (LAMP-2) and the red (CSPα) channels within the boxed area. The graph also shows the Pearson correlation between (*CSPα, red*) and Calnexin (*green*) and Giantin-1 (*green*) (See Supplemental Fig. 1C,D). (**D**) Representative Western blot of LAMP-1 and CSPα of the total cell homogenates (T) and lysosome enriched (L) fractions of murine fibroblasts. (**E**) Representative high magnification merged (*overlap, yellow*) pictures of N2A cells stained for endogenous CSPα (*CSPα, red*) and LAMP-2 (*LAMP-2, green*). Graph shows the co-localization index (Pearson correlation) between the green (LAMP-2) and the red (CSPα) channels both in the soma and neurites (See Supplemental Fig. 1E) within the boxed area. (**F**) Representative Western blot of LAMP-1 and CSPα of the total cell homogenates (T) and lysosome enriched (L) fractions of N2A cells. (**G**) Representative high magnification merged (*overlap, yellow*) pictures of primary cortical neurons stained for endogenous CSPα (*CSPα, red*) and LAMP-2 (*LAMP-2, green*). Graph shows the co-localization index (Pearson correlation) between the green (LAMP-2) and the red (CSPα) channels both in the soma and neurites (See Supplemental Fig. 1F) within the boxed area. (**H**) Representative Western blot of LAMP-1 and CSPα monomers (M-CSPα) and CSPα aggregates (Aggregates) from the total cell homogenates (T) and the lysosome-enriched fraction (L) of primary fibroblasts from two age-matched controls, two asymptomatic CSPα mutation carriers and from CSPα-deficient fibroblasts stably expressing CSPα-p.L115R (CSPα−/− p.L115R) or both CSPα-WT plus CSPα-p.L115R (CSPα−/− p.L115R + WT).

CSPα exhibits weak plasma membrane localization, diffuse cytoplasmic distribution and strong perinuclear immunoreactivity in both normal human dermal fibroblasts (Fig. 3A) and primary fibroblasts from a wild type mouse transduced with an empty lentiviral vector (Fig. 3C). Dual immunostaining revealed that a fraction of endogenous CSPα co-localizes with the lysosome (Fig. 3A,C) but does not co-localize with ER or Golgi markers (Supplementary Fig. 1C,D). In a differentiated neuron-like cell (N2A), CSPα exhibits a punctate pattern through the neurites and an enrichment in synaptic boutons, which is compatible with its localization in vesicles. In both N2A cells and primary cortical neurons, a fraction of endogenous CSPα co-localizes with LAMP2 in the soma, neurites and synaptic boutons (Fig. 3E,G and Supplemental Fig. 1E,G). Subcellular fractionation showed that a significant proportion of CSPα co-sediments with another lysosome marker (LAMP1) in three different cell types (Fig. 3B,D,F). Mutant CSPα-p.L115R aggregates are also found in the lysosome-enriched fractions of primary fibroblasts from CSPα mutation carriers and from CSPα-deficient fibroblasts stably expressing CSPα-p.L115R or expressing both CSPα-WT plus CSPα-p.L115R (Fig. 3H).

### Lysosome dysfunction caused by CSPα mutation p.L115R is recapitulated *in vitro*

**Figure 4:**
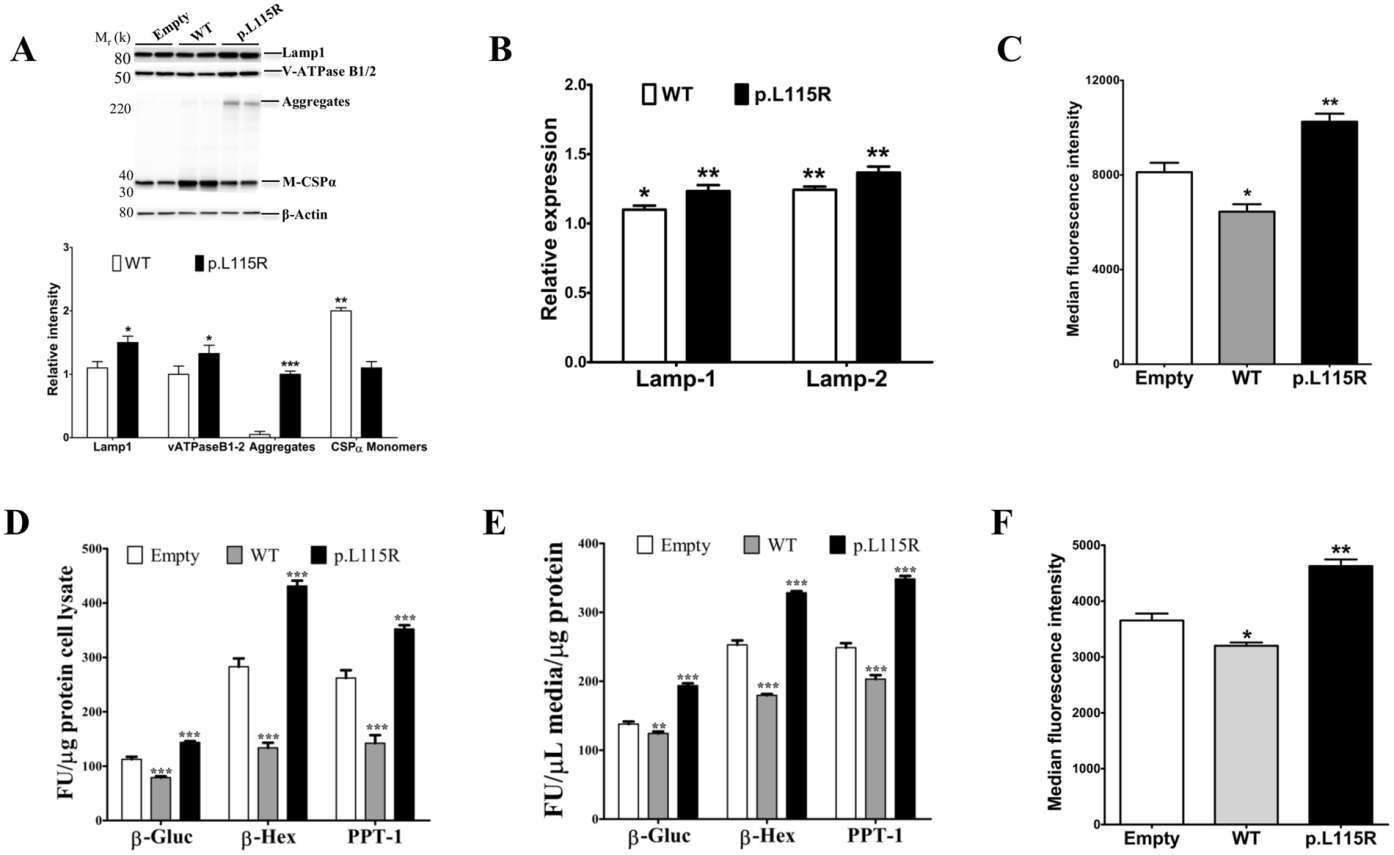
Lysosome dysfunction depends on the CSPα.pL115R mutation. (**A**) Representative Western blots illustrate the expression of LAMP-1, V-ATPase B1/2, CSPα monomers (M-CSPα) and CSPα aggregates (Aggregates) in fibroblasts from a wild-type mouse stably expressing nothing (Empty), CSPα-WT (WT) or CSPα-p.L115R (p.L115R). The histogram shows the quantification of LAMP-1, V-ATPase B1/2, CSPα monomers (M-CSPα) and CSPα aggregates (Aggregates) detected by immunoblot relative to protein levels in cells expressing the Empty vector. Proteins are normalized to β-actin. (**B**) The histogram shows the quantification of the transcript levels of LAMP-1 and LAMP-2 in fibroblasts from a wild-type mouse stably expressing CSPα-WT (WT) or CSPα-p.L115R (p.L115R) normalized to levels found in cells expressing nothing (Empty). Values represent the mean ± S.E. of three independent experiments *p = 0.02; **p = 0.01. (**C**) Lysotracker signal in primary fibroblasts from a wild-type mouse stably expressing nothing (Empty), CSPα-WT (WT) or CSPα-p.L115R (p.L115R). Values represent the mean ± S.E. of three independent experiments *p = 0.03; **p = 0.01. (**D**) Graph shows the activities of the lysosomal enzymes, β-gluc, β-Hexa and PPT-1 measured in cell homogenates of a wild-type mouse stably expressing nothing (Empty), CSPα-WT (WT) or CSPα-p.L115R (p.L115R). Enzymatic activity was normalized to the total intracellular protein. Values represent the mean ± S.E. of three independent experiments compared to the levels of cells transduced with empty vector ***, p ≤ 0.001 . (**E**) Graph shows the lysosomal enzyme activities of β-gluc, β-Hexa and PPT-1 measured in the culture medium. Enzymatic activity was normalized to the total intracellular protein. Values represent the mean ± S.E. of three independent experiments compared to the levels of cells transduced with empty vector ***, p ≤ 0.001. (**F**) Non-permeabilized surface LAMP-1 levels analyzed by flow cytometry using LAMP1-1DB4 (anti-intraluminal) in primary fibroblasts from a wild-type mouse stably expressing nothing (Empty), CSPα-WT (WT) or CSPα-p.L115R (p.L115R). Values represent the mean ± S.E. of three independent experiments compared to the levels of cells transduced with empty vector *, p = 0.02; **, p = 0.0047.

Primary fibroblasts from a wild-type mouse stably expressing CSPα-p.L115R from a lentiviral vector resulted in accumulation of CSPα aggregates (Fig. 4A), higher levels of lysosome-associated proteins (V-ATPase B1/2 and LAMP-1) (Fig. 4A) and greater transcript levels of LAMP-1 and LAMP-2 (Fig. 4B) than found in cells transduced with the empty vector. Significantly higher levels (~26%, p = 0.01) of LysoTracker signal (Fig. 4C), significant elevations of intracellular and secreted lysosomal enzymes (Fig. 4D,E) and significant increases in the amount of lysosome markers translocated to the plasma membrane (Fig. 4F) were found in cells expressing CSPα-p.L115R compared to the empty vector.

In contrast, overexpression of CSPα-WT results in no changes in the levels of lysosomal proteins (Fig. 4A) but higher levels of transcripts of LAMP-1 and LAMP-2 (Fig. 4B). In addition, there were significant reductions (~20%, p = 0.03) of LysoTracker signal (Fig. 4C), intracellular and secreted lysosomal enzymes (Fig. 4D,E) and the amount of lysosome markers translocated to the plasma membrane (Fig. 4F) compared to cells expressing the empty vector.

### Both CSPα-WT and CSPα-p.L115R are required for lysosome dysfunction

**Figure 5:**
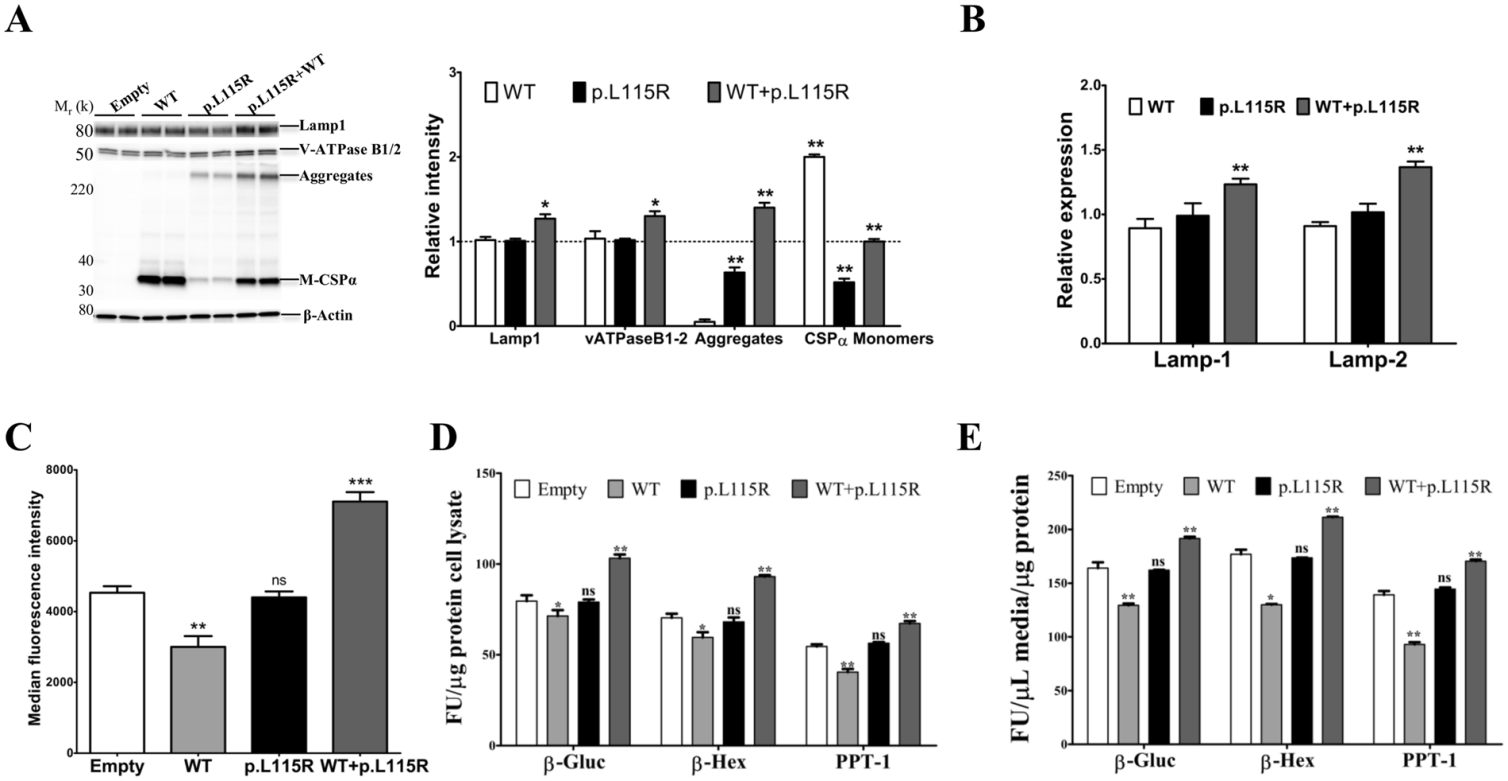
CSPα mutation p.L115R alone failed to cause lysosome dysfunction. (**A**) Representative Western blots illustrate the expression of LAMP-1, V-ATPase B1/2, CSPα monomers (M-CSPα) and CSPα aggregates (Aggregates) in fibroblasts from a CSPα-deficient mouse stably expressing nothing (Empty), CSPα-WT (WT), CSPα-p.L115R (p.L115R) or both CSPα-WT plus CSPα-p.L115R (WT+p.L115R). The histogram shows the quantification of LAMP-1, V-ATPase B1/2, CSPα monomers (M-CSPα) and CSPα aggregates (Aggregates) detected by immunoblot relative to protein levels in cells expressing the Empty vector. Proteins are normalized to β-actin. (**B**) The histogram shows the quantification of the transcript levels of LAMP-1 and LAMP-2 in fibroblasts from a CSPα-deficient mouse stably expressing CSPα-WT (WT), CSPα-p.L115R (p.L115R) or both CSPα-WT plus CSPα-p.L115R (WT+p.L115R) normalized to levels found in cells expressing the Empty vector. Values represent the mean ± S.E. of three independent experiments compared to the levels of cells transduced with empty vector **, p ≤ 0.01. (**C**) Lysotracker signal in primary fibroblasts from a CSPα-deficient mouse stably expressing nothing (Empty), CSPα-WT (WT), CSPα-p.L115R (p.L115R) or both CSPα-WT plus hCSPα-p.L115R (WT+p.L115R). Values represent the mean ± S.E. of three independent experiments compared to the levels of cells transduced with empty vector **, p = 0.01; ***, p = 0.001. (**D**) Graph shows the activity of the lysosomal enzymes, β-gluc, β-Hexa and PPT-1 measured in cell homogenates. Enzymatic activity was normalized to the total intracellular protein. Values represent the mean ± S.E. of three independent experiments compared to the levels of cells transduced with empty vector *, p < 0.05; **, p < 0.01. (**E**) Graph shows the activity of the lysosomal enzymes, β-gluc, β-Hexa and PPT-1 measured in the culture medium. Enzymatic activity was normalized to the total intracellular protein. Values represent the mean ± S.E. of three independent experiments compared to the levels of cells transduced with empty vector *, p < 0.05; **, p < 0.01.

To date, lysosomal dysfunction has not been reported in CSPα-deficient mice^24^. To isolate the effects of CSPα-p.L115R, fibroblasts from the CSPα-deficient mouse were transduced with an empty lentiviral vector or lentiviral vectors expressing CSPα-WT, CSPα-p.L115R or both CSPα-WT and p.L115R. Higher levels of CSPα aggregates, lysosome associated proteins (V-ATPase B1/2 and LAMP-1) (Fig. 5A) and transcript levels of LAMP-1 and LAMP-2 were found in cells co-expressing CSPα-p.L115R and CSPα-WT (Fig. 5B). Co-expression of CSPα-p.L115R and CSPα-WT was the only condition that resulted in significant elevations (~56%, p = 0.001) of LysoTracker signal (Fig. 5C) and both intracellular and secreted lysosomal enzymes (Fig. 5D,E).

Expression of CSPα-p.L115R alone resulted in the accumulation of CSPα aggregates but lower levels of CSPα monomers compared to cells expressing CSPα-WT alone (Fig. 5A). There were no significant differences in proteins (Fig. 5A) or transcript levels of lysosomal markers (Fig. 5B), levels of LysoTracker signal (Fig. 5C) or intracellular and secreted lysosomal enzymes between fibroblasts expressing CSPα-p.L115R or the empty vector (Fig. 5D,E).

Overexpression of CSPα-WT did not change the levels of proteins (Fig. 5A) or transcripts of lysosomal markers (Fig. 5B) but resulted in a significantly reduced (~34%, p = 0.01) LysoTracker signal (Fig. 5C) and intracellular and secreted lysosomal enzymes (Fig. 5D,E) compared to cells expressing either CSPα-p.L115R or empty vector.

### Wild-type and mutant CSPα.pL115R and its aggregates are degraded by the macroautophagy/lysosomal pathway

**Figure 6:**
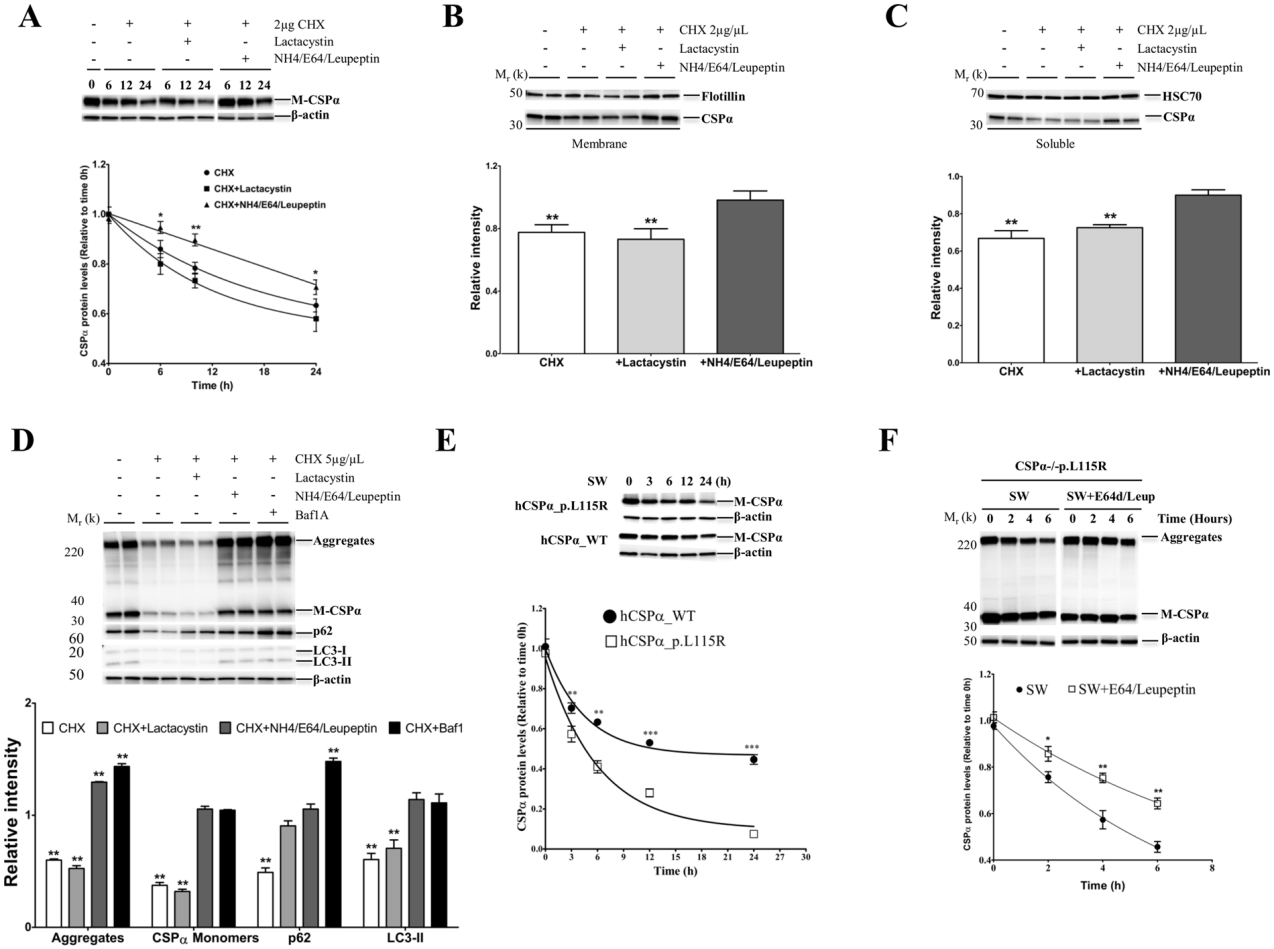
Autophagy-Lysosomal degradation of CSPα and CSPα.pL115R aggregates. (**A**) Representative Western blot displaying the levels of endogenous CSPα monomers (M-CSPα) in N2A cells treated with CHX, CHX plus Lactacystin or CHX plus NH4 + E64d + Leupeptin for 6, 12 and 24 hours. The graph shows the quantification of CSPα monomers detected by immunoblot. Values represent the mean ± S.E. of three independent experiments compared to the levels of cells treated with CHX alone *, p = 0.05; **, p = 0.01. (**B**) Representative Western blot of CSPα monomers (CSPα) in the membrane-enriched fraction (Membrane) in N2A cells treated with CHX, CHX plus Lactacystin or CHX plus NH4 + E64d + Leupeptin for 24 hours. The histogram shows the quantification of CSPα monomers detected by immunoblot relative to protein levels in untreated N2A cells. Proteins are normalized to Flotillin. (**C**) Representative Western blot of CSPα monomers (CSPα) in the cytosolic/soluble (Soluble) fraction in N2A cells treated with CHX, CHX plus Lactacystin or CHX plus NH4 + E64d + Leupeptin for 24 hours. The histogram shows the quantification of CSPα monomers detected by immunoblot relative to protein levels in untreated N2A cells. Proteins are normalized to HSC70. (**D**) Representative Western blot displaying the levels of CSPα monomers (M-CSPα) and CSPα aggregates (Aggregates) in fibroblasts from a CSPα-deficient mouse stably expressing CSPα-p.L115R treated with CHX, CHX plus Lactacystin, CHX plus NH4 + E64d + Leupeptin or CHX plus Bafilomycin A1 for 24 hours. The histogram shows the quantification of p62, LC3-II, CSPα monomers (M-CSPα) and CSPα aggregates (Aggregates) detected by immunoblot relative to protein levels in untreated cells. Proteins are normalized to β-actin. (**E**) Representative Western blot showing the levels of CSPα monomers (M-CSPα) in primary fibroblasts from a CSPα-deficient mouse stably expressing hCSPα-WT or hCSPα-p.L115R under serum withdrawal (SW) for 3, 6, 12 and 24 hours. The graph shows the quantification of CSPα monomers detected by immunoblot relative to protein levels in untreated cells at time = 0 h. Proteins are normalized to β-actin. (**F**) Representative Western blot showing the levels of CSPα monomers (M-CSPα) and CSPα aggregates (Aggregates) in fibroblasts from a CSPα-deficient mouse stably expressing CSPα-p.L115R (CSPα−/− p.L115R) under Serum withdrawal (SW) or SW + E64d + Leupeptin (SW + E64d/Leup) for 2, 4 and 6 hours. The graph shows the quantification of CSPα aggregates detected by immunoblot relative to protein levels in untreated cells at time = 0 h. Proteins are normalized to β-actin.

In order to understand the degradative pathway of wild-type CSPα, N2A cells were treated with the protein synthesis inhibitor (Cycloheximide, CHX) plus proteasome (Lactacystin) or lysosome (NH4/E64d/Leupeptin) inhibitors and collected at 6, 12 and 24 hours. There is a significant reduction in the levels of endogenous CSPα in cells treated with CHX alone or CHX plus Lactacystin (Fig. 6A). In contrast, lysosome inhibitors significantly prevented the degradation of endogenous CSPα compared with either CHX alone or CHX plus lactacystin (Fig. 6A). The effect of lysosome inhibitors was greater in the membrane-bound (Fig. 6B) compared to the soluble fraction of endogenous CSPα (Fig. 6C). Similarly, CSPα-deficient cells stably expressing CSPα-p.L115R treated with lysosome or macroautophagy inhibitors (Bafilomycin A1) prevented the degradation of CSPα-p.L115R monomers and induced a further accumulation of mutant CSPα-p.L115R aggregates (Fig. 6D). The levels of p62 and LC3-II proteins confirm the activation or inhibition of the macroautophagy/lysosomal pathway (Fig. 6D).

To confirm that the macroautophagy/lysosomal pathway is the primary pathway of degradation of CSPα, CSPα-deficient fibroblasts stably expressing CSPα-WT treated with serum withdrawal (SW) for 24 hours exhibited a significant reduction (44%, p < 0.01) in CSPα-WT levels (Fig. 6E). In contrast, the levels of CSPα-p.L115R were barely detectable (10%, p < 0.01) after 24 hours under SW (Fig. 6E). These results suggest that the degradation rate of CSPα-p.L115R is faster than CSPα-WT (Fig. 6E).

Both CSPα-p.L115R monomers and aggregates were reduced under SW (Fig. 6F). However, the presence of lysosome inhibitors prevented the reduction of both CSPα-p.L115R monomers and aggregates (Fig. 6F) due to the SW.

Serum withdrawal induced a significant reduction in both monomeric and aggregated forms of CSPα in the cytosolic and membrane-bound fractions in CSPα-deficient cells stably expressing CSPα-p.L115R or both CSPα-WT plus CSPα-p.L115R (Supplementary Fig. 3B).

### Decreased AFSM and CSPα aggregates in response to pharmacological treatment

**Figure 7:**
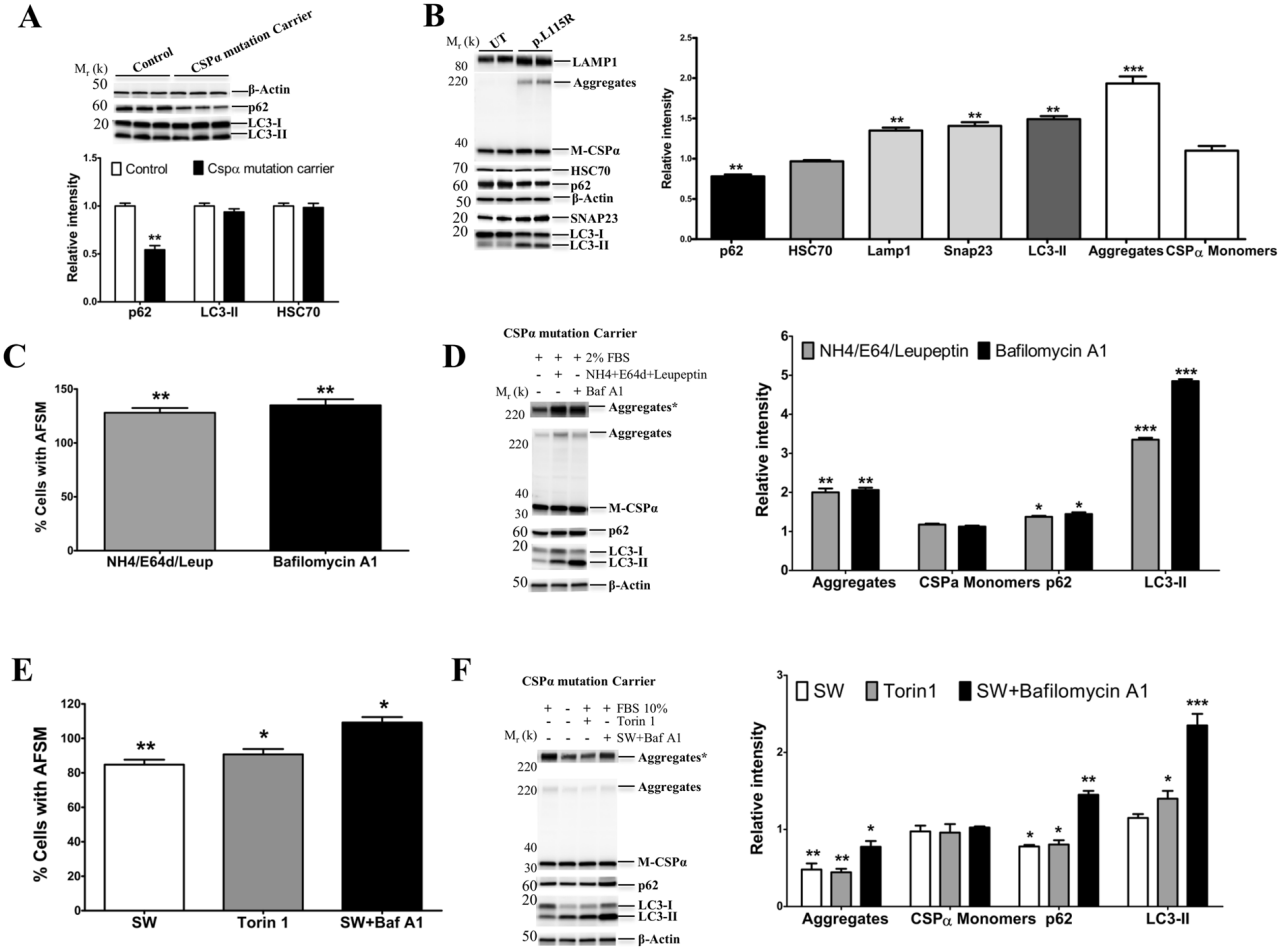
AFSM and CSPα aggregate accumulation are modulated by the autophagy-lysosome pathway. (**A**) Baseline Western blot of p62 and LC3-I/II in primary fibroblasts from an age-matched control and an asymptomatic CSPα mutation carrier. The histogram shows the quantification of p62 and LC3-II detected by immunoblot relative to protein levels in control cells. There are no changes in HSC70 levels.﻿ Proteins are normalized to β-actin. (**B**) Representative Western blot of Lamp1, CSPα monomers (M-CSPα) and CSPα aggregates (Aggregates), HSC70, p62, LC3-I/II and SNAP23 in untransduced (UT) and stably expressing CSPα-p.L115R (p.L115R) N2A cells. The histogram shows the quantification of p62, HSC70, LAMP1, SNAP23, LC3-I/II, Aggregates and CSPα monomers detected by immunoblot relative to protein levels in untrandusced (UT) N2A cells. Proteins are normalized to β-actin. (**C**) Quantitative analysis by flow cytometry of AFSM in primary fibroblasts from an asymptomatic CSPα mutation carrier maintained at 2% FBS for 6 days in culture and treated with NH4 + E64d + Leupeptin (NH4 + E64 + Leup) or Bafilomycin A1 relative to untreated cells. Values represent the mean ± S.E. of three independent experiments compared to the levels of untreated cells **, p < 0.01. (**D**) Representative Western blot of CSPα monomers (M-CSPα), CSPα aggregates (Aggregates), p62 and LC3-I/II in primary fibroblasts from an asymptomatic CSPα mutation carrier treated with NH4 + E64d + Leupeptin (NH4 + E64 + Leup) or Bafilomycin A1 (Baf A1) relative to untreated cells. Upper bands represent a longer exposure of the aggregates (Aggregates*). The histogram shows the quantification of CSPα monomers (M-CSPα), CSPα aggregates (Aggregates), p62 and LC3-I/II detected by immunoblot relative to protein levels in untreated cells. Proteins are normalized to β-actin. (**E**) Quantitative analysis by flow cytometry of AFSM in primary fibroblasts from an asymptomatic CSPα mutation carrier maintained at 10% FBS for 6 days in culture and treated with 0% serum (SW), Torin-1 or SW plus Baf A1 for 24 hours compared to the levels of untreated cells *, p < 0.05; **, p < 0.01. (**F**) Representative Western blot of CSPα monomers (M-CSPα-), CSPα aggregates (Aggregates), p62 and LC3-I/II in primary fibroblasts from asymptomatic CSPα mutation carrier treated with 0% FBS, Torin-1 or SW plus Baf A1 for 24 hours. Upper bands represent a longer exposure of the aggregates (Aggregates*). The histogram shows the quantification of CSPα monomers, CSPα aggregates (Aggregates), p62 and LC3-I/II detected by immunoblot relative to protein levels in untreated cells *, p < 0.05; **, p < 0.01. Proteins are normalized to β-actin.

Human fibroblasts typically exhibit high levels of LC3-II levels under basal conditions^26^. There were no significant differences in LC3-I or LC3-II levels under basal conditions in fibroblasts from AD-ANCL patients compared to controls (Fig. 7A). However, there was a reduction in p62 levels in asymptomatic CSPα mutation carriers compared to controls (Fig. 7A).

N2A cells stably expressing CSPα-p.L115R accumulate CSPα-p.L115R aggregates, have increased levels of LAMP1, SNAP23 and LC3-II proteins and lower p62 protein levels compared to untransduced N2A cells (Fig. 7B).

Cells from CSPα mutation carriers treated with lysosome and macroautophagy inhibitors have significant increases in AFSM accumulation (Fig. 7C). Both lysosome and macroautophagy inhibitors also led to an increase in mutant CSPα aggregates and increased levels of both LC3-II and p62, confirming that the autophagy flux is intact in fibroblasts from AD-ANCL patients (Fig. 7D).

In contrast, fibroblasts from asymptomatic CSPα mutation carriers treated with serum withdrawal (SW) or Torin 1 (macroautophagy activator) for 24 hours resulted in a significant reduction in AFSM (Fig. 7E) and mutant CSPα-p.L115R aggregates (Fig. 7F). The presence of macroautophagy inhibitors prevented the reduction of both AFSM (Fig. 7E) and CSPα aggregates (Fig. 7F) due to the SW. The reduction in p62 levels confirms the activation of the macroautophagy pathway.

### NtBuHA synergizes with SW to reduce both CSPα-p.L115R/CSPα-WT aggregates and AFSM

**Figure 8:**
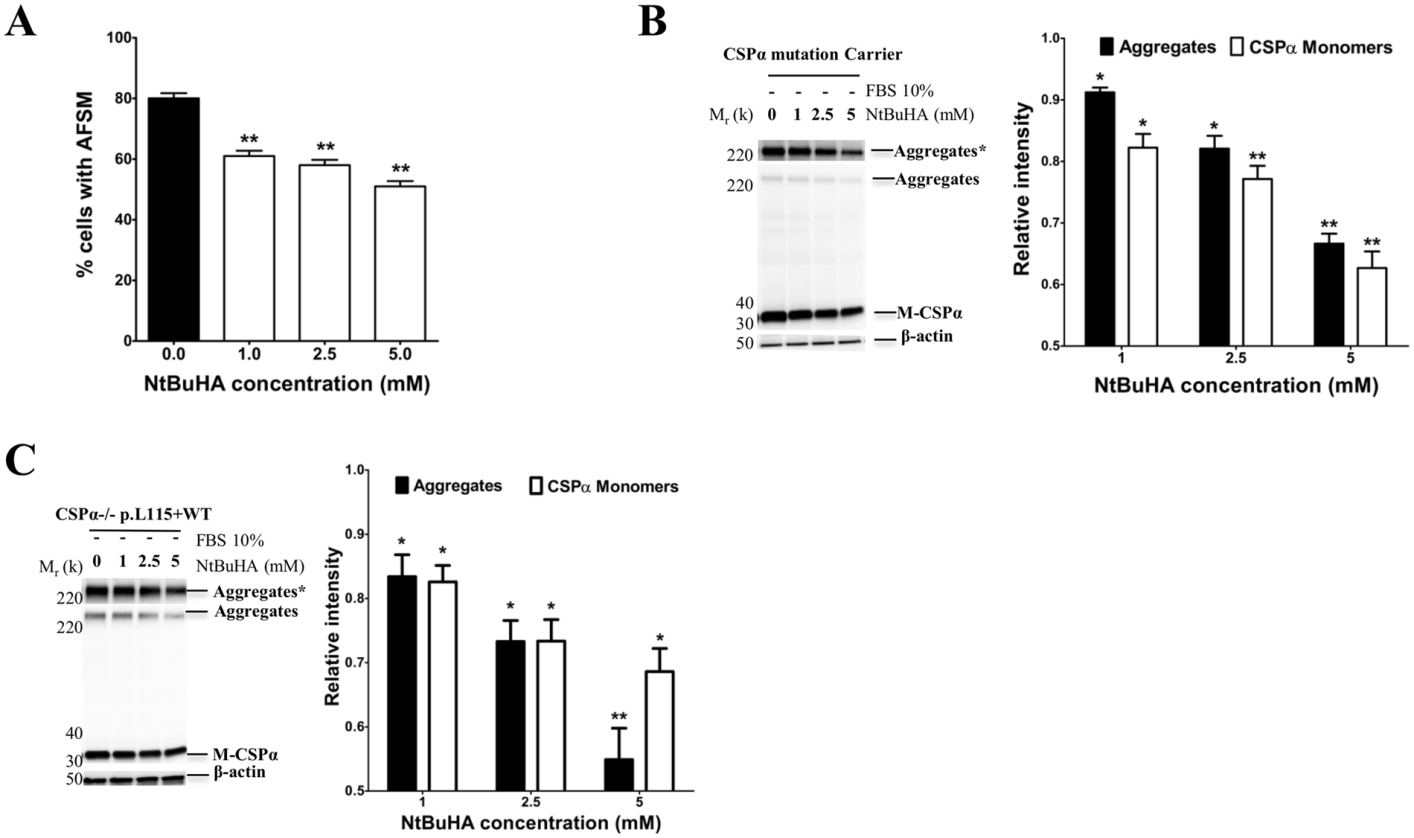
NtBuHA enhances the reduction of CSPα aggregates and AFSM induced by serum withdrawal. (**A**) Quantitative analysis by flow cytometry of AFSM in primary fibroblasts from an asymptomatic CSPα mutation carrier treated with increasing doses of N-(tert-Butyl)hydroxylamine (NtBuHA) for 24 hours in the absence of serum compared to the levels of untreated cells **, p < 0.01. (**B**) Representative Western blot of CSPα monomers (M-CSPα) and CSPα aggregates (Aggregates) in cultured fibroblasts from an asymptomatic CSPα mutation carrier treated with increasing doses of NtBuHA for 24 hours in absence of serum. Upper bands represent a longer exposure of the aggregates (Aggregates*). The histogram shows the quantification of CSPα monomers (M-CSPα) and CSPα aggregates (Aggregates) detected by immunoblot relative to protein levels in untreated cells. Proteins are normalized to β-actin. (**C**) Representative Western blot of CSPα monomers (M-CSPα) and CSPα aggregates (Aggregates) in homogenates from CSPα-deficient mouse fibroblasts stably expressing both CSPα-WT plus CSPα-p.L115R (CSPα−/− p.L115R + WT) treated with increasing doses of NtBuHA for 24 hours in the absence of serum. The histogram shows the quantification of CSPα monomers (M-CSPα) and CSPα aggregates (Aggregates) detected by immunoblot relative to protein levels in untreated cells. Proteins are normalized to β-actin.

It was recently shown that CSPα aggregation depends on its palmitoylation status^25^ and that brain tissue from terminal AD-ANCL patients exhibit aberrant PPT-1 activity^16^. PPT-1 is a lysosomal hydrolase that removes thioester-linked fatty acyl groups such as palmitate from cysteine residues in proteins including CSPα *in vitro* ^16^. Recently, a non-toxic derivative of hydroxylamine which mimics PPT-1 action, N-(tert-Butyl) hydroxylamine (NtBuHA), reduced the AFSM in lymphocytes and fibroblasts from patients with infantile NCL^27^. Here, NtBuHA failed to change the palmitoylation status of CSPα monomers, as evidenced by a lack of a shift in mobility of M-CSPα on a Western blot (Fig. 8B,C). However, the reduction of AFSM, CSPα monomers and CSPα aggregates in primary fibroblasts from asymptomatic CSPα mutation carriers induced by serum withdrawal was augmented by NtBuHA in a dose-dependent manner (Fig. 8A,B). This finding is replicated in CSPα-deficient cells stably expressing CSPα-p.L115R plus CSPα-WT treated with NtBuHA under serum withdrawal (Fig. 8C).

## Discussion

We demonstrate that primary dermal fibroblasts recapitulate features of AD-ANCL *in vitro* including AFSM accumulation and CSPα-p.L115R/CSPα-WT aggregates found in the brains of AD-ANCL patients. In addition, both CSPα-p.L115R/CSPα-WT aggregates and AFSM are susceptible to pharmacological intervention *in vitro*. The macroautophagy activators (serum withdrawal and ATP-competitive mTOR kinase inhibitor, Torin 1) reduced both CSPα-p.L115R/CSPα-WT aggregates and AFSM. This reduction is synergized by the addition of N-tert-Butyl Hydroxylamine. These results open a new avenue for possible treatment of this fatal disease. However, it is not clear, why there is no change in the palmitoylation status CSPα monomers in cells treated with NtBuHA. CSPα aggregation is induced and maintained by palmitoylation^25^. Here, we used the NtBuHA concentrations reported by Sarkar C *et al*. that are able to cleave thioester linkages in [^14^C]palmitoyl~CoA without affecting the cell survival^28^. These NtBuHA concentrations reduced the ASFM and CSPα aggregates in a synergistic manner with macroautophagy activation in primary fibroblasts from CSPα mutation carriers. However, CSPα is one of the most highly palmitoylated proteins; its cysteine string domain contains 12–15 cysteine residues, each one of which acts as a palmitoylation site^24^. Thus is possible that the concentration and the time of exposure to NtBuHA used in this study do not affect the palmitoylated form of CSPα monomers but preferentially CSPα aggregates. Interestingly, Nosková L *et al*. reported that CSPα monomers were detectable in their specific AD-ANCL patient brain samples only in the presence of a depalmitoylating agent (Hydroxylamine)^5^. Two independent groups suggested that chemical depalmitoylation solubilized the CSPα aggregates, which resulted in an increase in the pool of CSPα monomers^5, 25^. The preferential effect of NtBuHA on CSPα aggregates makes it more attractive from a therapeutic perspective as we show it affects the pathogenic aggregates without affecting the neuroprotective monomer form of CSPα.

The levels of AFSM are inversely correlated with behavioral changes and response to therapies and directly correlated with severity of clinical and neuropathological presentation of the disease in at least two murine NCL models^29–31^. Recently, AFSM in cultured lymphocytes and fibroblasts from INCL patients was successfully used in a cell-based screening that resulted in the identification of a small molecule drug that is currently being used in clinical trials for INCL patients^27^. AFSM represents a rare opportunity for therapeutic development as it exhibits several targetable aspects such as specificity for NCLs, is an easily quantifiable phenotype, involves several steps in its formation and degradation and is a biomarker that correlates with clinical disease^29^. These characteristics provide multiple potential sites for pharmacological intervention. Resolution of AFSM detected in cultured cells from NCL patients can be a reliable indicator of treatment efficacy for some LSDs^32^. Thus, we propose the use of AFSM as a quantitative fluorometric trait in cellular models for testing therapy intervention for AD-ANCL.

CSPα’s long half-life^18^, absence of canonical ubiquitylation sites^19^, localization at the autophagosome and its responsiveness to treatment with Rapamycin^33^ suggest that it might be degraded by the autophagy-lysosomal pathway. In addition, published reports on the degradation of CSPα by the UPS are contradictory^15, 20, 21^. There are only two papers addressing the UPS degradation of CSPα in wild-type primary neurons^15^ or neuron-like cell lines^20^ with opposite results. In addition, Sambri *et al*. reported that proteasome inhibition prevented the degradation of CSPα (mostly depalmitoylated in this model) to a greater extent than lysosome inhibitors in primary neurons from a murine model of LSD (MPS-IIIA)^15^. Here, we demonstrate that endogenous CSPα co-localizes with lysosomal proteins and is found in lysosome-enriched fractions from three different cell types, including a neuron-like cell line and primary cortical neurons. These findings are consistent with previous reports by independent groups using various sub-cellular fractionation techniques^11–14^. In addition, we provide evidence that the ALP is primarily responsible for the degradation of endogenous CSPα-WT, mutant CSPα-p.L115R and its aggregates in both fibroblasts and a neuron-like cell line. All together, these results suggest that the contribution of the UPS and ALP to the degradation of endogenous CSPα-WT may depend on the specificity of the UPS and ALP inhibitor used, cell type, CSPα palmitoylation status and cell state (diseased vs normal condition).

We also show that the turnover rate of CSPα-p.L115R by the ALP is faster than CSPα-WT. These findings, along with the intrinsic propensity of CSPα-p.L115R to self-assemble into insoluble aggregates account for the reduced CSPα monomer levels found in fibroblasts and brain homogenates of AD-ANCL patients^6, 7, 22, 25^. This further supports the loss-of-CSPα function in AD-ANCL due to a haploinsufficiency as one of the components of the disease mechanism. In the absence of co-chaperone CSPα, the resulting misfolded partners are degraded by the proteasome^34^. Brain homogenates from terminal AD-ANCL patients exhibit significant reductions in SNARE-complex - forming presynaptic proteins (e.g SNAP-25)^17^. Interestingly, there is a compensatory increase in proteasome activity in the brains of AD-ANCL patients (Supplementary Fig. 3C). Therefore, a high rate of degradation of CSPα’s partners may contribute to the cellular pathophysiology of AD-ANCL and explain the massive neuronal and synaptic loss found in terminal AD-ANCL patients^17, 22^. Alternatively, with reduced CSPα levels, misfolded CSPα clients might contribute to the formation and accumulation of AFSM.

CSPα may play a role in lysosome-membrane fusion events. This hypothesis is supported by previous studies showing that CSPα is critical for maintaining levels of SNARE proteins^35^ and by our data showing that both mutant CSPα and overexpression of wild-type CSPα affect the levels of lysosome enzymes in the media and the amount of lysosomal markers in the plasma membrane. Synaptosomal associated protein of 23 kDa (SNAP-23) is a ubiquitously expressed SNARE protein that belongs to the SNAP-25 family^36^. SNAP-23 is present in the plasma membrane of many types of cells and mediates exocytosis of secretory vesicles and lysosome-membrane fusion events^36^. A recent report suggested that CSPα is a key mediator in the exocytosis of tau, α-synuclein and TDP-43 through a SNAP23-mediated exocytosis^37^. Our data show that primary fibroblasts from asymptomatic CSPα mutation carriers and N2A cells stably expressing CSPα-p.L115R exhibit higher SNAP-23 levels than controls and consequently, an elevation in the amount of secreted lysosomal enzymes. Therefore, our results support a role of CSPα in lysosome exocytosis.

Maintenance of the lysosomal compartment depends on continuous fusion of late endocytic structures accompanied by fission events^38^. The small GTP binding protein Rab7 plays an important role in the maturation of autophagosomes and lysosome biogenesis^39^. Primary fibroblasts from asymptomatic CSPα mutation carriers exhibit a reduction in Rab7 levels compared to controls. This suggests that CSPα-p.L115R induces an increase in the rate of autophagosome-lysosome fusion. In addition, p62 levels are reduced in CSPα mutation carriers compared to controls, which suggests an increase in autophagy. However, there are no significant changes in steady-state level LC3-I or LC3-II protein. In the presence of lysosome or macroautophagy inhibitors there is an increase in the levels of p62 and LC3-II proteins suggesting that autophagic flux is intact in fibroblasts from asymptomatic CSPα mutation carriers. However, the persistent elevation of LC3-II under macroautophagy activation after 24 hours suggests a block in the fusion of autophagosomes and lysosomes^40^ or dysfunctional lysosomes. This is supported by a reduction in p62 and an increase in steady-state LC3-II, LAMP1 and SNAP23 levels found in N2A cells stably expressing CSPα-p.L115R. These results are also consistent with an elevation in transcription factor EB (TFEB), which regulates lysosomal biogenesis and function, reported in brains of AD-ANCL patients^16^ and the significant changes in the transcript levels of lysosomal proteins reported here in response to CSPα-p.L115R mutant.

AD-ANCL is caused by mutations in a synaptic protein and how this results in the massive neurodegeneration found in terminal AD-ANCL patients remains to be clarified^23, 24^. In addition to being a synaptic protein, we show here that CSPα is also a lysosomal protein. Our data show that CSPα-p.L115R is not sufficient to cause AFSM accumulation or lysosomal dysfunction. In contrast, both wild-type and CSPα-p.L115R are required to result in an AD-ANCL phenotype (AFSM accumulation and lysosomal dysfunction) *in vitro*. These data suggest that CSPα plays a role in lysosome function. In a brain from an asymptomatic CSPα mutation carrier there is accumulation of AFSM with minimal to no changes in the levels of CSPα^17^. Thus, we hypothesize that AD-ANCL is a protein aggregation disease where the pathogenic mechanism is correlated with the presence of CSPα-p.L115R/CSPα-WT aggregates and its subsequent effects on the ALP function. These findings support a gain-of-function mechanism for CSPα mutations leading to AD-ANCL. However in light of the reduced CSPα levels found in terminal AD-ANLC patients, we hypothesize that the most likely disease mechanism involves a combination of the loss of CSPα’s neuroprotective function and gain-of neurotoxic function resulting from CSPα-p.L115R/CSPα-WT aggregates.

## Methods

### Cell Culture

Primary subdermal fibroblasts from AD-ANCL patients and controls were collected according to a Washington University in St Louis Human Subject Committee approved protocol and grown in RPMI-1640 medium. Primary subdermal fibroblasts from CSPα -deficient mice (B6;129S6-Dnajc5tm1Sud/J, Jackson Laboratory, Maine, USA) were isolated from new born animals and grown in DMEM. All animal procedures were approved by the Institutional Animal Studies Committee at Washington University School of Medicine and were in accordance with the guidelines of the National Institutes of Health.

All cell lines were supplemented with 10% heat inactivated FBS, 10 mM HEPES buffer, MEM non-essential amino acid solution, 1mM sodium pyruvate, 1% penicillin/streptomycin under 5% pCO2 at 37 °C. Neuro-2A (N2A) cells (gift from Dr Celeste Karch at WUSTL) were maintained in 50% Dulbecco’s Modified Eagle’s Medium (DMEM) and 50% Opti-MEM supplemented with 5% FBS, 1% L-Glutamine and 1% penicillin/streptomycin.

### Lentivirus Preparation and transductions

A human *DNAJC5* cDNA clone was obtained from Origene (SC305246–20) in a pCMV6-XL6 vector. The mutation (c.344T > G) was engineered using a site-directed mutagenesis kit (QuikChange II (Agilent Technology, Santa Clara, CA, USA). Wild-type and mutant cDNAs were subcloned into a pLenti-III-PGK Vector (Applied Biological Materials Inc, Richmond, Canada). The resultant lenti vectors along with plasmids coding for VSV-G, Gag-Pol, and Rev were transfected into HEK-293T packaging cells as previously described^41, 42^. Viral supernatant was collected according to previously published protocols^41^. Cells were cultured with unconcentrated viral supernatants for 24 hours and cells were selected with 5 μg/ml of puromycin (Sigma-Aldrich, St. Louis, MO) for four weeks. The titer levels of transgene expression were measured by quantitative real-time PCR using human *DNACJ5* gene specific primers: hDNAJC5_F, 5′-AGTCATTGTACCACGTCCTTG-3′; hDNAJC5_R, 5′-TCTCCTTAAACTTGTCCGCG-3′.

### Lysosomal enzyme activity

One hundred µg of tissue from each brain region from three terminal AD-ANCL patients and three neurodegenerative pathology-free controls^17^ was homogenized in buffer containing 10 mM Tris (pH 7.5), 150 mM NaCl, 1 mM dithiothreitol, and 0.2% Triton X-100 and centrifuged at 14,000 rpms for 1 min at 4 °C. Following centrifugation, the supernatant was removed and used for PPT-1, β-gluc, and β-Hexa enzyme assays as previously described^43, 44^. Cell lysates and medium from was collected after 6 days in culture.

### Proteasome activity assay

The proteolytic activity of the proteasome (catalytic core of the 26S proteasome) was evaluated in brain homogenates (occipital lobe) from three terminal AD-ANCL patients and three neurodegenerative pathology-free controls using the 20S proteasome activity kit (APT 280; Millipore), following the manufacturer’s instructions. Briefly, 5 μg of whole brain protein extract were incubated in duplicates in the provided buffer with 50 μM fluorophore-linked peptide substrate (LLVY-7-amino-4-methylcoumarin [AMC]) for 30 min at 60 °C. Proteasome activity was measured by quantification of relative fluorescent units from the release of AMC using a 380/460 nm filter set in a fluorometer (Synergy™ H4 Hybrid Multi-Mode Microplate Reader; Biotek). A solution of the 20S proteasome subunit (1:100 dilution) and the proteasome inhibitor lactacystin were used as controls for the assay. An AMC standard curve was performed with each experiment.

### Immunofluorescence

Cultured cells were fixed in ice-cold freshly-prepared 4% paraformaldehyde in PBS, permeabilized in 0.25% Triton-X 100 (Sigma–Aldrich, MO, USA) for 10 min, and blocked by incubation in 10% goat serum^45^. Cells were incubated in primary antibody CSPα (AB1576; Millipore) 1:500, Calnexin (MAB3126, Millipore) 1:500, Giantin (ALX-804–600; Enzo life science) 1:1000, LAMP-1 (sc-19992, Santa Cruz Biotechnology) 1:500 or LAMP-2 (H4B4, Hybridoma Bank) 1:500, for 16 h at 4 °C, followed by secondary antibodies conjugated to Alexa-488 (1:1000) or Alexa-568 (1:1000) (Molecular Probes, Eugene, Oregon) for 2 h at room temperature. DAPI (4′,6-Diamidino-2-Phenylindole, Dihydrochloride) (Molecular Probes®, Eugene, Oregon) was used as counterstaining for the nuclei. Controls were stained omitting the primary or secondary antibody. Imaging was performed on a confocal microscope (LSM 700; Carl Zeiss, Jena, Germany) using a Plan-Apochromat 63×/1.4 oil differential interference contrast objective (Carl Zeiss) at room temperature with Zen 2009 software. Images were acquired using ZEN 2009 software (Zeiss) exported as TIFF images and brightness and contrast were adjusted using Image J. All images are single confocal slices with a maximum projection of a confocal Z-stack (performed using ZEN 2009 software). For quantitative analysis of the colocalization of CSPα and LAMP2, Pearson’s colocalization coefficient (*R*) was calculated with ZEN 2009 software (Carl Zeiss) for pixels with intensities above background in a cell from two-color images of multiple cells.

### Treatment of Cells with Cycloheximide (CHX) plus Lysosome inhibitors

Cells were grown in 6-well plates to 50–80% confluence and maintained in 10% heat inactivated FBS. Twenty-four hours after plating, cells were incubated with 2 or 5 µg/mL CHX (fresh CHX was added every 12 hours) plus 20 mM NH4Cl plus 100 µM Leupeptin plus 10 µg/mL E64D or 10 µM Lactacystin^46^ or 100 nM Bafilomycin A1 (Dissolved in DMSO, Sigma, St. Louis). Cycloheximide, NH4Cl, E64D and Leupeptin were dissolved in water. Lactacystin (BML-PI104) was purchased from Enzo Life Technology, NY.

### Treatment of Cells with Lysosome and proteasome inhibitors

Cells were grown in 6-well plates to 80% confluence and maintained in 2% heat inactivated FBS. Six days after plating, cells were treated with 20 mM NH4Cl plus 100 µM Leupeptin plus 10 µg/mL E64D or 100 nM Bafilomycin A1. Six days after plating, cells at 10% FBS were treated with SW; 250 nM Torin 1 or carrier (water or DMSO) as a control. NH4Cl (A5666), E64D (E3132), Bafilomycin 1A (B1793) and Leupeptin (L2884) were purchased from Sigma, St. Louis, MO. Torin 1 (4247) was purchased from Tocris Bioscience, UK.

### Treatment of Cells with N-(tert-Butyl) hydroxylamine

Cells were grown in 6-well plates to 80% confluence and maintained in 2% heat inactivated FBS. Six days after plating cells were incubated with 0.1–5 mM N-(tert-Butyl) hydroxylamine (479675, Sigma-Aldrich)^27^ for one hour followed by 23 hours of serum starvation (SW).

### Lysosomal fraction isolation

Purification of lysosomal fractions from cultured cells was performed using the Lysosome Enrichment Kit for Tissue and Cultured Cells (Thermo Fisher Scientific, Waltham, MA) following the manufacturer instructions. Briefly, 50 mg of pelleted cells were homogenized in Lysosome Enrichment Reagent A using a dounce homogenizer (30 strokes), followed by the addition of the same volume of Lysosome Enrichment Reagent B. The nuclei, cell debris, and mitochondria were removed by a 10-min centrifugation at 1,000 × g at 4 °C. A “crude lysosomal fraction (CLF)” containing the lysosomes, mitochondria, peroxisomes, endoplasmic reticulum and microsomes was obtained by centrifugation of supernatants at 20,000 × g for 20 min at 4 °C. Lysosomes were purified from the CLF by the ultracentrifugation (150,000 × g for 4 h in a Beckman SW 60 Ti Rotor, Swinging Bucket) in a discontinuous density gradient (17–30%) of iodixanol (OptiPrep). Immediately after centrifugation each fraction was probed for mitochondria, peroxisomes, and Golgi and ER proteins as well as for the presence of the lysosomal membrane proteins LAMP-2, LAMP-1 and Hexosaminidase by Western blot (Supplementary Fig. 2A).

### Surface LAMP1 Assay

Surface LAMP1 assay was performed as previously described^47^. Briefly, cells were trypsinized, collected in FACS buffer and incubated with anti-rat LAMP1–1DB4 (sc-19992, Santa Cruz Biotechnology) at 4 °C for 30 min. Cells were washed in PBS. Anti-LAMP1-1D4B–treated cells were further incubated with Alexa-488 conjugated anti–rat secondary antibodies (Molecular Probes, Invitrogen) for 30 min at room temperature and fixed in 1% paraformaldehyde (PFA). Finally, cells were analyzed on Gallios flow cytometer (Beckman Coulter). Data were analyzed using FlowJo (Tree Star).

### Immunoblotting

Cells and brain tissue were lysed in radioimmune precipitation assay (RIPA) buffer (50 mM Tris-HCl, pH 7.4, 150 mM NaCl 1% Nonidet P-40, 0.25% sodium deoxycholate) plus 1X phenylmethanesulfonylfluoride (PMSF) and 1X Protease Inhibitor Cocktail (P2714, Sigma, St. Louis, MO) for 10 min on ice and then spun at 14,000 rpm for 10 min at 4 °C. Protein was subjected to electrophoresis and transferred to PDVF membrane (BIO-RAD, Hercules, CA). The primary antibodies were diluted as follows: CSPα (ADI-VAP-SV003-E; Enzo life science) 1:20000, HSC70/HSP73 (ADI-SPA-816; Enzo life science) 1:1000, LAMP-1 (H4A3, Hybridoma Bank) 1:50000, LAMP-2 (H4B4, Hybridoma Bank) 1:50000, Saposin D (Kindly provided by Prof. Sandhoff, University of Bonn, Bonn, Germany) 1:500, LAMP-1 (1D4B, sc-19992, Santa Cruz Biotechnology) 1:2000, CLN1 (sc-130726, Santa Cruz Biotechnology) 1:100, V-ATPase B1/2 (sc-55544, Santa Cruz Biotechnology) 1:1000, Synaptosomal-associated protein 23 (TS-19, S2194, Sigma-Aldrich) 1:5000, p62 (Anti-SQSTM1, 2C11, Abnova) 1:1000, LC3 (NB100-2331, Novus Biologicals) 1:5000, Flotillin (C-7, sc-133153, Santa Cruz Biotechnology) 1:5000, β-Actin (A1978, Sigma-Aldrich) 1:5000. The membranes were then incubated with the secondary antibodies, horseradish peroxidase-conjugated anti-mouse or anti-rabbit IgG (KPL, Gaithersburg, MD) diluted 1:2000 in 4% nonfat dry milk PBS-T for 1 h at room temperature. Signals were visualized using Lumigen ECL Ultra (TMA-6) (Lumigen, Southfield, MI). Densitometric semi-quantification was performed using ImageJ software (National Institutes of Health).

### Flow cytometry

Cells were harvested, washed once in PBS, re-suspended at ∼1 × 10^6^ cells/ml in FACS buffer (PBS, 1 mM EDTA, 2% FBS). AFSM data from 2 × 10^4^ cells per condition were recorded and analyzed by flow cytometry. All flow cytometry data were collected on a Gallios flow cytometer (laser 488 nm, Channels FL1, FL2 and FL10) (Beckman Coulter). Collected data were analyzed using FlowJo (Tree Star, Ashland, OR).

### Cytosolic and membrane-bound fraction isolation

Cells from three 150 mm petri dishes at 100% confluence were trypsinized, pelleted, re-suspended in HES homogenization buffer (0.32 M sucrose, 20 mM HEPES, 1 mM EDTA, pH 7.4, plus protease inhibitors) and homogenized with a Dounce homogenizer. Cytosolic (fraction S2) and membrane-bound (fraction P2) proteins were extracted as previously described^17, 48^.

### Analysis of Clinical Samples

The Institutional Review Board at the Washington University in Saint Louis School of Medicine approved the study. Prior to their participation, written informed consent was reviewed and obtained from family members. The Human Research Protection Office approval number (201104178). The neuropathological findings of AD-ANCL patients were previously published^2, 6^. *DNAJC5* mutation identification and screening was published by Benitez *et al*.^6, 17^.

### Statistical analyses

All data are shown as means ± SEM. Two-way ANOVA with Bonferroni post-test was used to determine the difference between genotypes and treatments and the possible interactions of each. For comparison of two groups, Student’s unpaired two-tailed t test was used. Data were analyzed using GraphPad Prism, version 5.00 (San Diego, CA).

## Electronic supplementary material

Supplemental Material
