# Supplementary material for: Primary fibroblasts from CSPα mutation carriers recapitulate hallmarks of the adult onset neuronal ceroid lipofuscinosis
Source: Sci Rep. 2017 Jul 24;7:6332. doi: 10.1038/s41598-017-06710-1 (PMC5524943; doi:10.1038/s41598-017-06710-1)
Supplement: Supplementary file 1 — Supplemental Material [file 41598_2017_6710_MOESM1_ESM.pdf]

**Primary fibroblasts from CSPα mutation carriers recapitulate hallmarks of the adult onset neuronal ceroid lipofuscinosis**

**Bruno A. Benitez[1*], Mark S. Sands[1,2,3]**

[1]Department of Medicine, Washington University School of Medicine, St. Louis, MO, 63110, USA. [2]Department of Genetics, Washington University School of Medicine, St. Louis, MO, 63110, USA. [3]Hope Center for Neurological Disorders, Washington University School of Medicine, St. Louis, MO, 63110, USA.

**Supplementary Material**

***Supplementary Figure 1.***

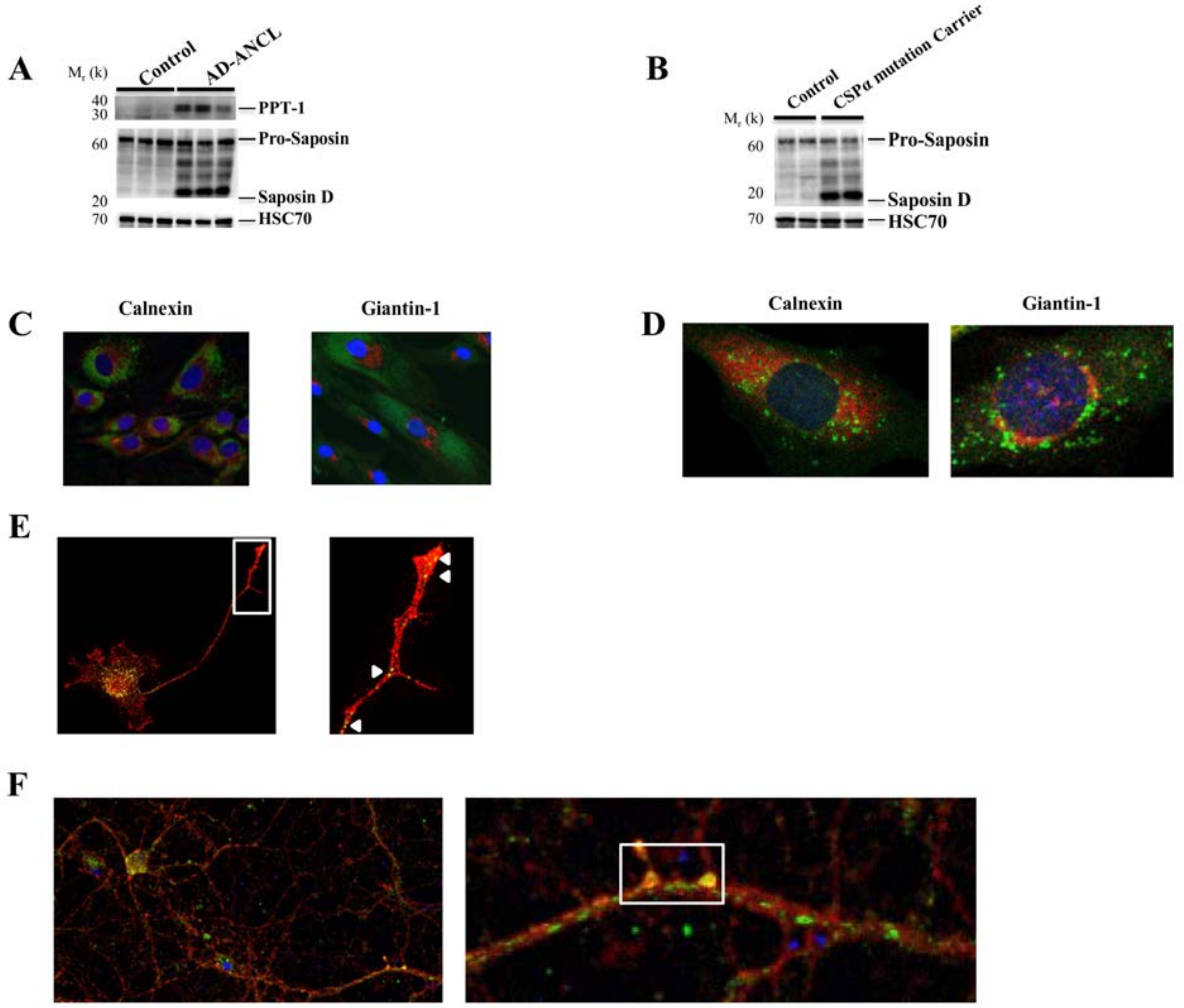

***A,*** Representative Western blots showing the expression of Pro-Saposin, Saposins D and PPT-1 in occipital lobe from three controls and three AD-ANCL patients.

***B,*** Representative Western blots showing the expression of Pro-Saposin and Saposins D in fibroblasts of controls and asymptomatic CSPα mutation carriers. Soluble proteins are normalized to HSC70.

***C,*** Representative pictures of co-immunostaining of normal human primary fibroblasts. Nuclear staining (*blue*), Endogenous CSPα (*green*) and Calnexin (*ER,red*).

***D,*** Representative pictures of co-immunostaining of normal murine primary fibroblasts. Nuclear staining (*blue*), Endogenous CSPα (*green*) and Giantin-1 (*Golgi, red*).

***E,*** Representative pictures of co-immunostaining of N2A cells soma and neurites. Endogenous CSPα (*red*) and Lamp-1 (*Lysosome, green*).

***F,*** Representative pictures of co-immunostaining of primary cortical neurons soma and neurites. Nuclear staining (*blue*), Endogenous CSPα (*red*) and Lamp-1 (*Lysosome, green*).

***Supplementary Figure 2.***

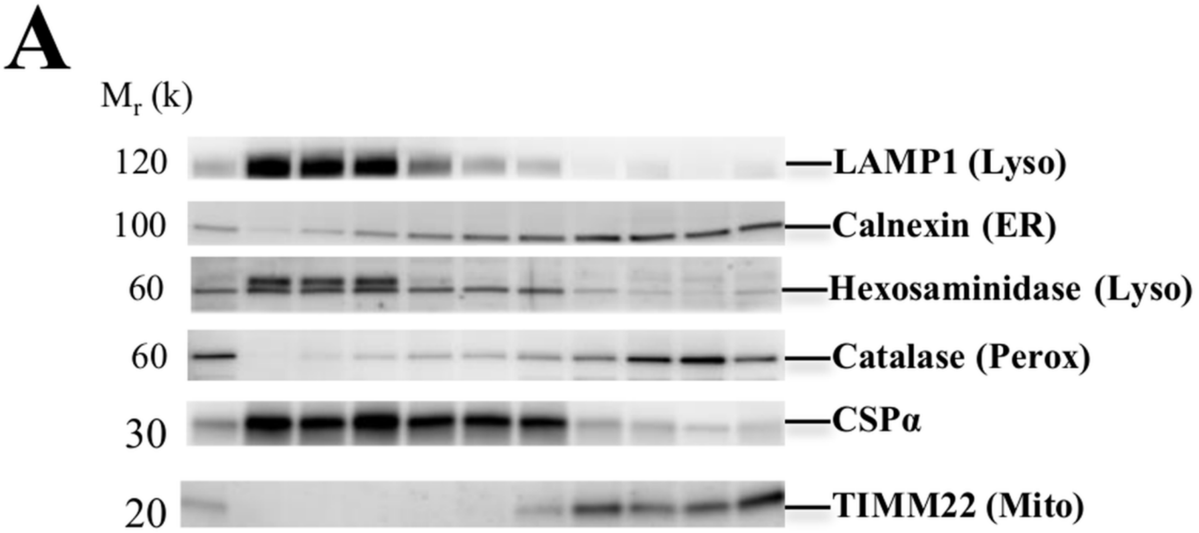


***A,*** Representative Western blots showing the expression of organelle markers LAMP-1 (lysosome), Calnexin (ER), Hexosaminidase (lysosome), Catalase (peroxisome), and TIMM22 (Mitochondria).

***Supplementary Figure 3.***

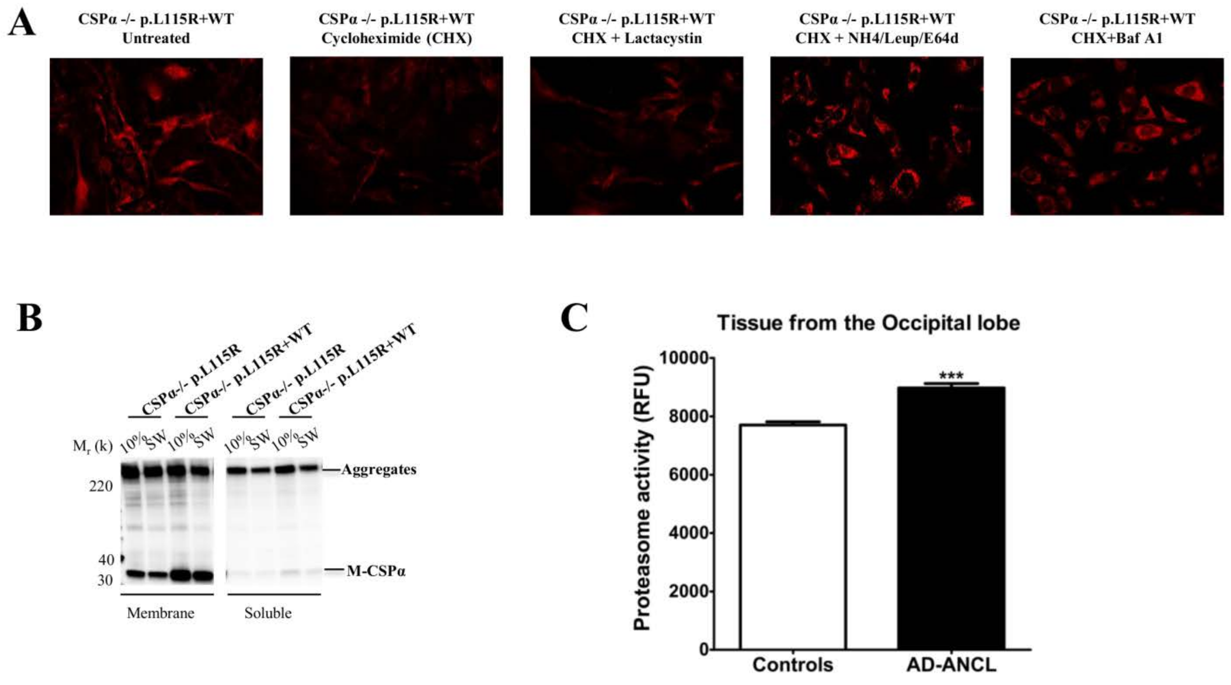


***A,*** Representative pictures of immunostaining of CSPα-deficient mouse stably expressing both hCSPα-WT plus hCSPα-p.L115R (CSPα-/- p.L115R+WT) treated with CHX 5 µg/mL or CHX plus Lactacystin or CHX plus NH4+E64d + Leupeptin or CHX plus Baf A1 for 24 hours. Endogenous CSPα (*red*).

***B,*** Representative western blots of CSPα monomers (M-CSPα-) and CSPα aggregates (Aggregates) in the membrane-enriched fraction (Membrane) and cytosolic/soluble (Soluble) in fibroblasts from a CSPα-deficient mouse stably expressing hCSPα-p.L115R (p.L115R) or both hCSPα-WT plus hCSPα-p.L115R (CSPα-/- p.L115R+WT) treated with SW (0% FBS) for 24 hours.

***C,*** Graph shows proteasome activity in the occipital lobe from AD-ANCL patients and in control individuals. Values represent the mean ± S.E. of three independent experiments. ***, p = 0.0005 using Student's t test.
